# An Instrument to Facilitate Skin-Grafting

**Published:** 1872-03

**Authors:** 


### An Instrument to Facilitate Skin-Grafting.

An instrument designed to facilitate the process of skin-grafting was brought into use at St. Bartholomew's Hospital in the early part of last March. It consists of a pair of curved scissors, which are provided on their concave surface with bent forceps. These are controlled by a lever which descends with the separation of the blades, and rises when they are brought together. The movements of these several parts are so concerted that the forceps meet between, and just below, the blades, immediately before the closure of the latter, and then rise between them to such a height that whatever they have seized will be divided from its attachment when the blades actually meet. Thus the whole process of seizing a small portion of the skin, separating, and raising it, can be almost simultaneously performed with one hand. The size of the severed piece of skin is proportionate to the force with which the forceps are pressed against the surface from which it is to be removed.

This contrivance was suggested to Mr. Ferguson, of Giltspur-street, early in the present year, by Mr. Crips, a student of St. Bartholomew's. It has since been in constant use at the hospital, and is very favourably spoken of. Mr. Pollock also has, we believe, expressed his approval of it."—"*Braithwait's Retrospect.*"

